# A low latency and low power indirect topology for on-chip communication

**DOI:** 10.1371/journal.pone.0222759

**Published:** 2019-10-02

**Authors:** Usman Ali Gulzari, Sarzamin Khan, Muhammad Sajid, Sheraz Anjum, Frank Sill Torres, Hessam Sarjoughian, Abdullah Gani

**Affiliations:** 1 Department of Electrical Engineering, The University of Lahore, Islamabad, Pakistan; 2 Department of Electrical Engineering, COMSATS University Islamabad, Wah Campus, Wah Cantt, Pakistan; 3 Department of Electrical and Computer Engineering, University of Western Ontario, London, Ontario, Canada; 4 Department of Computer Science, COMSATS University, Islamabad, Wah Campus, Wah Cantt, Pakistan; 5 German Aerospace Center, Institute for the Protection of Maritime Infrastructures, Bremerhaven, Germany; 6 Arizona Center for Integrative Modeling & Simulation, Arizona State University, Tempe, United States of America; 7 Faculty of Computing and Informatics, Universiti Malaysia Sabah, International Campus Labuan, WP Labuan, Malaysia; Wroclaw University of Science and Technology, POLAND

## Abstract

This paper presents the Hybrid Scalable-Minimized-Butterfly-Fat-Tree (H-SMBFT) topology for on-chip communication. Main aspects of this work are the description of the architectural design and the characteristics as well as a comparative analysis against two established indirect topologies namely Butterfly-Fat-Tree (BFT) and Scalable-Minimized-Butterfly-Fat-Tree (SMBFT). Simulation results demonstrate that the proposed topology outperforms its predecessors in terms of performance, area and power dissipation. Specifically, it improves the link interconnectivity between routing levels, such that the number of required links isreduced. This results into reduced router complexity and shortened routing paths between any pair of communicating nodes in the network. Moreover, simulation results under synthetic as well as real-world embedded applications workloads reveal that H-SMBFT can reduce the average latency by up-to35.63% and 17.36% compared to BFT and SMBFT, respectively. In addition, the power dissipation of the network can be reduced by up-to33.82% and 19.45%, while energy consumption can be improved byup-to32.91% and 16.83% compared to BFT and SMBFT, respectively.

## Introduction

The growing complexity of System-on-Chip (SoC) designs, characterized by an increasing amount of Processing Elements (PEs), requires intelligent solutions for the communication among the PEs. In response to this challenge, Networks-on-Chip (NoC) has been proposed. NoC is a new promising paradigm, which targets efficient communication between PEs [1]. It is based on packet switching and routing techniques in order to improve utilization of the on-chip interconnections, leading to enhanced network scalability and communication bandwidths as well as reduced power consumption and chip area utilization [2–4].

The topology of a NoC defines the organization of the connections of the routing nodes and, thus, has a high impact on the NoC’s characteristics [5]. The topology does not only impact the communication performance but also influences other parameters like power consumption, area, mapping strategy and architecture of routing nodes [6–9]. The design and selection of appropriate topology for a particular set of application play a key role in the efficient transmission of packets between any source/destination pair in the network [3]. Researchers try to design such topologies that can achieve reduced average power consumption while possessing the minimum average latency and maximum utilization of on-chip bandwidth [4]. Therefore, the principal challenge for all network topologies is the trade-off between performance and costs [10–11].

**Fig 1:**
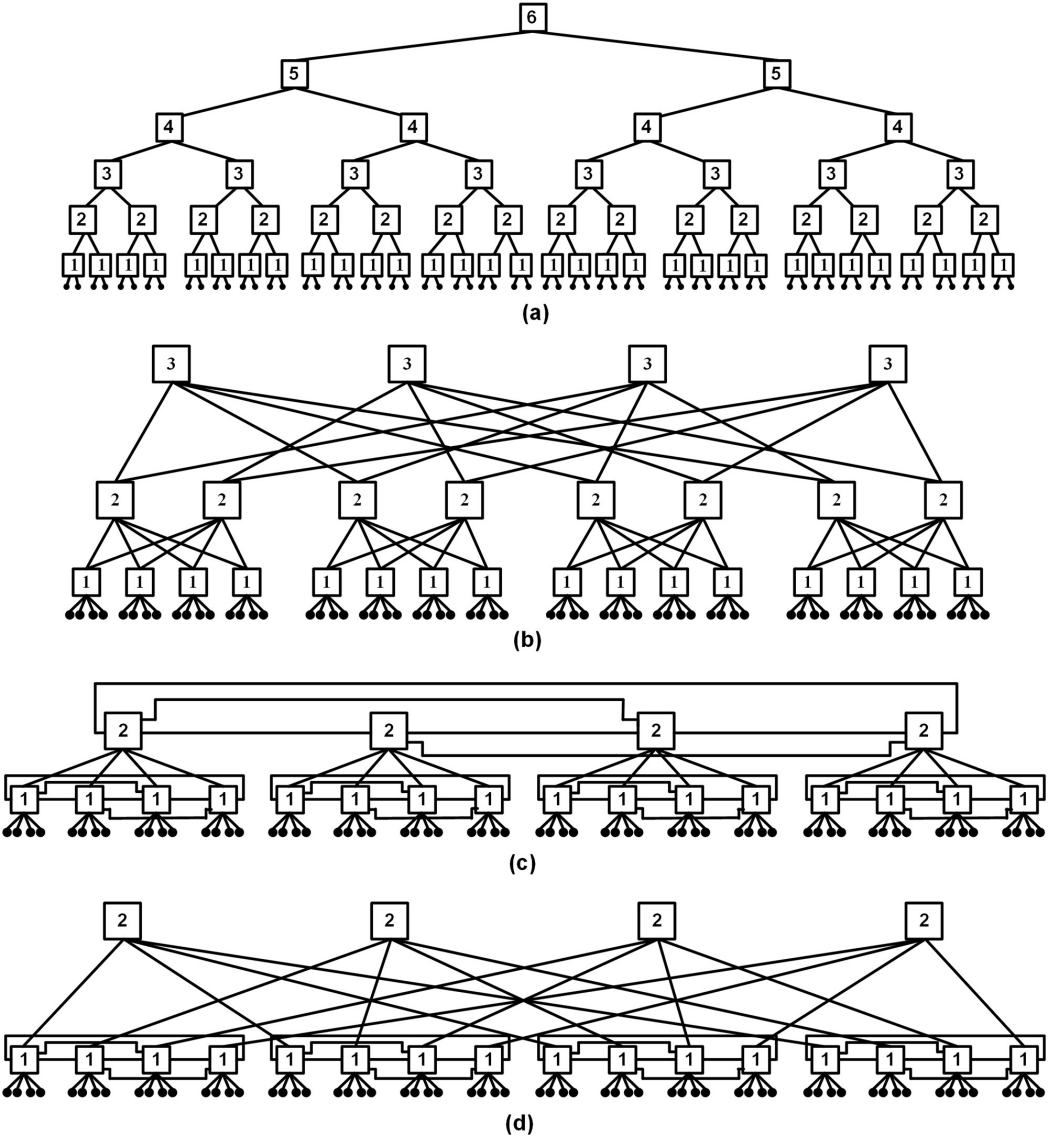
FT, BFT, SMBFT and H-SMBFT network topologies with 64 nodes with routers and links. (a) Fat-Tree network, (b) Butterfly-Fat-Tree network, (c) Scalable-Minimized-Butterfly-Fat-Tree network and, (d) Hybrid-Scalable-Minimized-Butterfly-Fat-Tree network.

This work proposes the Hybrid Scalable and Minimized-Butterfly-Fat-Tree (H-SMBFT) topology, which is an improved version of the Scalable and Minimized-Butterfly-Fat-Tree (SMBFT) and gives better performance in terms of average network latency, network area, and average power consumption of the network. The H-SMBFT reduces the number of levels in the network as compared to Extended-Butterfly-Fat-Tree interconnection (EFTI), Butterfly-Fat-Tree (BFT) and the Fat-Tree (FT) network [12–14]. The FT, BFT, and SMBFT take six, three and two levels respectively for 64 nodes network as shown in Fig 1(a)–1(c). Compared to its predecessors, the proposed H-SMBFT reduces the number of links, router complexity and the average length of the routing paths as shown in Fig 1(d). Consequently, the new topology leads to the lower amount of routing levels and reduced average network latency, area and power consumption.

All three topologies H-SMBFT, SMBFT, and BFT, have been compared in an extensive study based on two different types of NoC simulators [15–17]. We applied the established ORION 3.0 simulator [16] for the analysis of the required routers in terms of area utilization and power consumption. Next, we compared all three topologies regarding average latency as well as network power and energy consumption with the help of the widely used NoCTweak simulator [17]. All results have been determined for synthetic traffic traces and five real-time embedded application workloads. The simulation results indicate that H-SMBFT is an efficient NoC topology, with notable characteristics in terms of average latency and energy consumption.

The rest of the paper is organized as follows. Section-II highlights background work. Section-III focuses on the proposed topology and the architecture of the new topology H-SMBFT. Section-IV compares the characteristics of the proposed topology with BFT and SMBFT and presents simulation results. Finally, Section-V draws the conclusions.

## Background

Mesh is a well-known network topology using direct interconnections and is widely applied to on-chip communication due to its simple and regular design characteristics [18]. However, its simplicity comes at the cost of poor scalability. That means, increasing the size of a Mesh-based NoC system considerably degrades its performance, which is mainly based on the small bisection widths and large network diameters [19].

Amongst the several strategies for reducing the network diameter, concentration is the most promising one [20–22]. For example, the Concentrated-Mesh (C-Mesh) topology proposed in [20] reduces the network diameter by a factor of fourat the cost of higher router complexity. However, increasing the concentration factor increases quadratically the crossbar circuitry [21]and limits the concentration factor, and thus, reduces the network scalability. The flattened butterfly topology presented in [21] aims at circumventing this conflict by using high-radix routers [20]. However, each additional dimension leads to more complex routers which again restricts the scalability of the network [23].

The Fat-Tree (FT) topology is an alternative to Mesh and based on indirect interconnection derived from a binary tree [24]. It could be shown that FT has several advantages compared to Mesh, including improved bandwidth, reliability, scalability and regularity [14, 23–24]. The Extended-Butterfly-Fat-Tree-Interconnection (EFTI) [14] has an improved network diameter in comparison to the Butterfly-Fat-Tree (BFT) topology. The latter is an updated version of FT and received attention in recent applications [25–30]. The Scalable-Minimized-Butterfly-Fat-Tree (SMBFT) topology proposed in [12] is a minimized version of EFTI and BFT that reduces the number of routers, links and levels, and consequently, provides improved performance in the terms of average network latency and power consumption [12, 14].

## H-SMBFT topology

The proposed Hybrid Scalable-Minimized-Butterfly-Fat-Tree topology is a combination of both SMBFT and BFT. It applies a concentration factor of four and sibling links to reduce the number of network levels compared to BFT. Limiting the concentration factor to four reduces both the router complexity as well as the structural design of the network, leading to improved scalability.

Fig 1 depicts the structure of the FT, BFT, SMBFT and H-SMBFT implementations for a 64 node network. Square boxes represent the routers with their levels, and tiny black circles indicate the nodes of the network. The comparison reveals that the bottom level of H-SMBFT is identical to SMBFT (see Fig 1(c) and 1(d)), while the upper levels are interconnected similarly to the BFT network (see Fig 1(b) and 1(d)). Compared to SMBFT, the bottom level of H-SMBFT provides improved connectivity among the nodes and reduces the number of network levels. In the H-SMBFT version, all levels, except the bottom one, apply 5-port routers, while the SMBFT implementation uses the more complex and costlier 8-port routers.

In H-SMBFT, one router link connects to a parent router, and four links are used to connect to the router’s children. In the bottom level, each router has three additional sibling links that interlink to sibling routers, enabling improved connectivity compared to the BFT topology.

### 3.1. H-SMBFT network design

During the design of an H-SMBFT network, routers are positioned at vertices and nodes at the leaves. Each node, which can be a node or a router, is represented with *(p*, *l)*, where *p* is the position and *l* indicates the level of the node. Each router *R(p*, *l)* has one parent port and four child ports. The total number of network levels of the design follows from:

$$Levels=({\text{log}}_{4}N)-1$$ (1)

Where N is the total number of nodes. The number of routers at the *l*^*th*^ level can be estimated by Eq 2:

$$routers=\frac{N}{4^{l}}$$ (2)

The position *p(i)* of the parent router of *R(i*,*1)* can be calculated via Eq 3:

p(i)=(imod4) (3)

Where *i* indicate the position of a router at *level l* and ranges from 0 to *routers-1* (see Eq (2)). Consequently, the parent router of each router *R(p(i)*, *l)* is *R(i mod 4*,*l+1)*. The routers at bottom *level 1* have three additional links that are connected to the router’s siblings *(Right*, *Left*, *and Next or Cross)*, with *(Ri*,*1)*, *(Li*,*1)* and *(Ni*,*1)* are the representations of the right, left and next or cross siblings, respectively. The positions *Ri*, *Ni* and *Li* are given by Eqs 4 to 6:

$$R_{i}=\left\lfloor \frac{i}{4}\right\rfloor \times 4+(i+1)\text{mod}\;4$$ (4)

$$N_{i}=\left\lfloor \frac{i}{4}\right\rfloor \times 4+(i+2)\text{mod}\;4$$ (5)

$$L_{i}=\left\lfloor \frac{i}{4}\right\rfloor \times 4+(i+3)\text{mod}\;4$$ (6)

### 3.2. H-SMBFTaverage distance

The average distance (***D*_*avg*_**) of a network follows from:

$$D_{avg}=\frac{1}{N^{2}}\sum_{n_{i}n_{j}\in N}D(n_{i,}n_{j})$$ (7)

**Fig 2:**
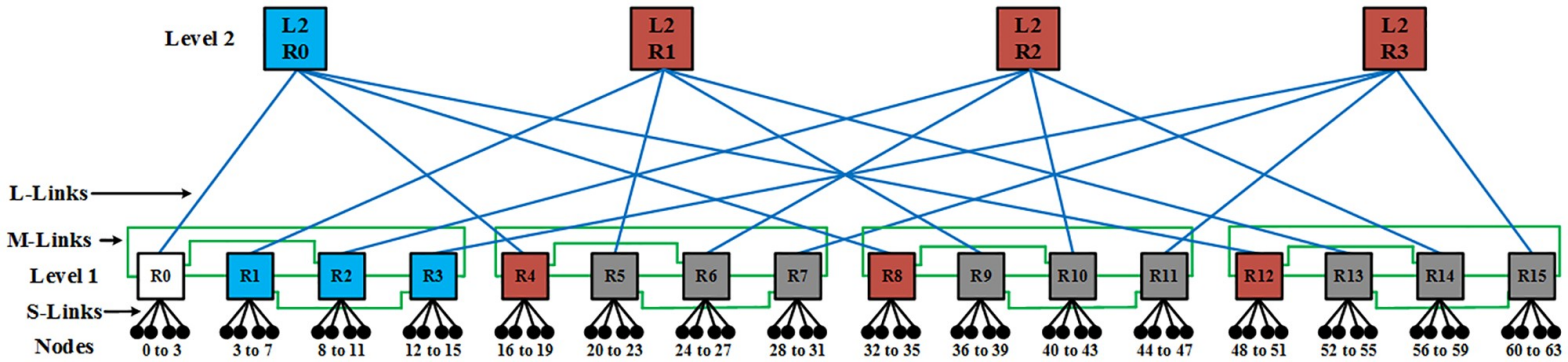
H-SMBFT network with interconnection links, router levels, and nodes. L-Link means Large-Links, M-Link is Medium-Links and S-Links indicates Small-Links.

Here *N* is the number of nodes, *n*_*i*_ and *n*_*j*_ are the source and destination nodes, respectively, and *D* is the shortest path for traversing data packet between ***n***_***i***_ and ***n***_***j***_ is given as unit hops [20,29]. Fig 2 depicts for the H-SMBFT the number of hops from the reference point node 0 to the remaining routers. Here, one hop is required to traverse a packet from node 0 to whit- colored routers and its linked nodes. Traversing a data packet from node 0 to blue-colored routers and linked nodes require two hops while traversing data to brown-colored routers requires three hops. Similarly, traversing data from node 0 to grey colored routers requires four hops.

Table 1 presents the distance in terms of hops from node 0 to all other nodes in BFT, SMBFT and H-SMBFT networks. The average distances computed using Eq 7 without considering router delay and errors comes out to be ***D***_***avg_BFT***_ = **4.38**, ***D***_***avg_SMBFT***_ = **3.63** and ***D***_***avg_H−SMBFT***_ = **3.44** for BFT, SMBFT and H-SMBFT networks having 64 nodes respectively. These values show that H-SMBFT can reduce the average distance by 5.2% and 21.4% compared to SMBFT and BFT network topologies respectively.

**Table 1:** Comparison of node distance for BFT, SMBFT and H-SMBFT networks.

**Node 0 distance to other nodes in BFT**				
Node 0 to	1 to 3	4 to 15	16 to 63	
Distance in hops	1	3	5	
**Node 0 distance to other nodes in SMBFT**				
Node 0 to	1 to 3	4 to 15	16 to 63	
Distance in hops	1	3	4	
**Node 0 distance to other nodes in H-SMBFT**				
Node 0 to	1 to 3	4 to 15	16 to19, 32 to 35, 48 to 51	20 to 31, 36 to 47, 53 to 63
Distance in hops	1	3	3	4

## Simulation results

We simulated all components of the network in order to obtain information about the area, power dissipation, network latency and energy dissipation [16–17]. Therefore, we employed the ORION 3.0 simulator [16] for estimation of area and power dissipation of all routers and links in the networks. The ORION 3.0 model achieves average estimation errors below 9.3% across microarchitecture and different RTL implementations of router components. We applied the NoCTweak [17] simulator for the scalability analysis of the BFT, SMBFT and H-SMBFT networks, by comparing average network latency and energy consumption. A notable characteristic of NoCTweak is that the tool not only considers the number of hops but also uses the post-layout synthesis results of all the router components for computations. Firstly, the RTL designs in Verilog of all router components were synthesized with a design compiler and placed and routed with Cadence SoC Encounter using a CMOS standard cell library of a 22nm technology. We defined links of 1000 μm between router modules and used the actual post-layout delay, throughput and energy values. Hence, the results obtained include the fidelity of real implementations.

When a processing element receives a packet, it subtracts the packet’s generating time (in the packet’s header flit) from the current simulation time to get the packet latency. The results obtained are therefore not merely dependent on the number of hops but also consider the post-layout results of delay, power and energy [17].

In the following, we detail the employed models for our analysis.

Network latency: Latency means the time taken by a header flit of a packet to traverse between any source-destination pair in the network. Latency also includes the time a packet waits at all intermediate buffers during its way from source to destination node due to the network congestion. The average network latency *L*_*avg*_ is therefore given by Eq (8):

$$L_{avg}=\frac{1}{N}\sum_{i=1\in N}\left(\frac{1}{N_{i}}\sum_{\forall j}L_{i,j}\right)$$ (8)

Where *L*_*i*_,_*j*_ is the packet latency of packet *j* and *N*_*i*_ is the number of packets received by node *i*, and *N* is the number of nodes in the network.

Power consumption: The power consumption of the network results from the activities of all components while running a certain traffic pattern. The average power *P*_*i*_ of router *i* is described by Eq (9):

$$P_{i}=\sum_{\forall j}\left[\alpha_{i,j}P_{act,j}+\left(1-\alpha_{i,j}\right)P_{inact,j}\right]$$ (9)

*P*_*act*,*j*_ and *P*_*inact*,*j*_ mean the active and inactive powerof component *j*, while α_*i*,*j*_ is the percentage of time the component *j* in router *i* is active (after the warm-up time).

Consequently, the average power of all the routers in the network is given by Eq (10):

$$P_{avg}=\frac{1}{N}\sum_{i=1\in N}P_{i}=\frac{1}{N}\sum_{i=1\in N}\sum_{\forall j}\left[\alpha_{i,j}P_{act,j}+(1-\alpha_{i,j})P_{inact,j}\right]$$ (10)

Network energy consumption: The average energy *E*_*avg*_ dissipated by each router during the simulation time *T*_*sim*_ after warm-up time *T*_*warm*−_*up* is given by Eq (11):

$$E_{avg}=P_{avg}(T_{sim}-T_{warm-up})$$ (11)

The average energy *E*_*P*_ dissipated per packet by each router is given by Eq (12):

$$E_{P}=\frac{E_{avg}}{N_{P}}=\frac{T_{sim}-T_{warm-up}}{N*N_{P}}\sum_{i=1\in N}\sum_{\forall j}[\alpha_{i,j}P_{act,j}+(1-\alpha_{i,j})P_{inact,j}]$$ (12)

Where *N*_*p*_ is the total number of packets transferred on the network and is given by *N*_*p*_ = ∑_i=1∊N_ *N*_*i*_.

Router and link area: The router area results from the sizes of the basic building blocks of the router, i.e. SRAM-FIFOs, crossbar and arbiter. For example, the area of an SRAM follows from Eqs (13) and (14):

$$L_{word-line}={fw.(w}_{cell}\;2(P_{r}-P_{w})d_{w}$$ (13)

$$L_{bit-line}={B.(h}_{cell}+(P_{r}-P_{w})d_{w}$$ (14)

Where *f*_*w*_, *w*_*cell*_, *P*_*r*_, *P*_*w*_, *d*_*w*_, *Bandh*_*cell*_, are flit width in bits, memory cell width, number of read ports, number of write ports, wire spacing, buffer size and memory cell height, respectively. Hence, the total area *Area*_*fifo*_ for a B-entry buffer results to:

$${Area}_{fifo}={BL_{word-line}.L_{bit-line}}_{cell}$$ (15)

The area of the remaining router components, i.e., crossbar and arbiter, can be estimated via its cell-level description and the information about cell sizes [17].

The area occupied by links is due to wires and repeaters. To estimate the area of repeaters the area of global wiring can be calculated from Eq (16):

$${Area}_{Link}={fw.{(w}_{s}+s_{w})+s}_{w}$$ (16)

where *Area*_*Link*_ denotes the wire area, *f*_*w*_ is the flit width in bits, and *w*_*s*_ the wire width and *s*_*w*_ the spacing computed from the width and spacing of the layer using a particular design style.

This section compares the characteristics of the proposed H-SMBFT topology in terms of average distance, router and link complexity. Further, simulation results for synthetic data and real-world examples are presented.

### 4.1. Router and link complexity

The ORION 3.0 simulator was used for the estimation of the power consumption and area utilization of the routers and links [16]. Therefore, we divided grouped all links into Large-Links (L-Link), Medium-Links (M-Link) and Small-Links (S-Link). The S-Links are used to connect nodes with routers in the first level. M-Links are used at level 1 to connect to sibling routers (R). Finally, L-Links connect routers of level l to level l-1. Fig 2 depicts all of these links for the H-SMBFT. We assumed the length of S-, M- & L-Links as 1000, 3000 and 8000 μm for the estimation of power consumption and area utilization. The different number of links type, router type, total area and total power for BFT, SMBFT and H-SMBFT with 64 node networks are shown in Table 2.

**Table 2:** Levels,router type, link type, power consumption and area utilization requirements for BFT, SMBFT, and H-SMBFT with 64 nodes using ORION 3.0 simulator.

Topology	No. of Levels	Router Type				Links Type			Total Power	Total Area
		4-Port	6-Port	7-Port	8-Port	S-Links	M-Links	L-Links	(Link + Router) mW	(Link + Router) mm^2^
BFT	3	4	24	-	-	64	32	16	3.95	14.27
SMBFT	2	-	-	4	16	64	24	22	3.65	12.24
H-SMBFT	2	4	-	-	16	64	24	16	3.30	11.26

Table 2 compares the required links and routers types for BFT, SMBFT and H-SMBFT networks with 64 nodes. The results indicate that H-SMBFT reduces the power consumption by 9.8% and 17.3% compared to SMBFT and BFT respectively. Also,H-SMBFT improves area utilization by 7.9% and 21.1% compared to SMBFT and BFT networks respectively. The error estimation was performed for the link, router power consumption and area utilization using the five seed t-student test [31]. The ±1.31, ± 1.64 and ± 1.82 percent error was found for the link and router power consumption w.r.t H-SMBFT, SMBFT, and BFT networks respectively. Similarly, the ±1.63, ± 1.87 and ± 1.96 percent error was recorded for the link and router area utilization w.r.t H-SMBFT, SMBFT and BFT networks respectively. The maximum recorded error value of ±1.96 shows the correctness of simulated results.

### 4.2. Simulation environment

We applied the NoCTweak simulator for the comparison of the topologies BFT, SMBFT and H-SMBFT regarding latency as well as power and energy consumption [17]. We choose NoCTweak because it considers the post-layout synthesized results of the entire router and link components. Furthermore, it is cycle-level accurate and also permits the integration of different network topologies. In order to compare the topologies, we integrated the BFT, SMBFT and H-SMBFT topologies and chose the simulation parameters as given in Table 3.

**Table 3:** Chosen parameter for all simulations under NoCTweak.

Parameters	Values
Technology	22 nm
Operating voltage	1.0 V
Clock frequency	1 GHz
Input buffer size	16 flits
Amount of virtual channels	8
Packet length (flit units)	150
Flit length	32 bits
Router	Five stage pipeline
Each simulation runs	100,000 cycles
Warm-up cycle time	20,000 cycles
Standard link length	1000 μm
Flit injection rate	0.01 to 0.9 flits/cycle/node

In an initial step, NoCTweak simulator synthesized the RTL designs of all router components with the Synopsys Design Compiler for a standard cell library in commercial CMOS technology. Next, the designs were placed and routed with the Cadence SoC Encounter, followed by the extraction of basic delay and power data. These data were then fed for estimation of power, energy, and delay based on the activities of components while running the selected traffic patterns. The standard link length between router modules is defined with 1000 μm. This length is set for each node by the NoCTweak simulator accordingly to the requirements of the design and follows the classification as Large-Links (L-Link), Medium-Links (M-Link) and Small-Links (S-Link) discussed in section 4.2. Two kinds of traffic patterns such as synthetic traffic traces and traces extracted from real-world application workloads were applied to all the networks for fair comparisons. The related results are presented in the following section.

### 4.3 Simulation results for synthetic data

The synthetic traffic patterns of Random, Hotspot, Transpose, Shuffle and Neighbor were applied for an initial comparison of all three topologies. Border critical cases are simulated by applying 100% traffic load and assigning a high priority to the extreme pairs in the 64 node networks.

Table 4 shows the absolute values and percentage savings of the H-SMBFT topology in terms of network power, energy, and average latency measurements.

**Table 4:** Network Power, Energy and Average Latency Improvements ofH-SMBFT with 64 nodes compared to BFT and SMBFT topologies for five different synthetic traffic traces using NoCTweak simulator.

Networks	BFT		SMBFT		H-SMBFT			
Average Network Power Consumption (uW)								
Applications	Power	Error ±	Power	Error ±	Power	Error ±	H-SMBFT% savings as compared to BFT	H-SMBFT% savings as compared to SMBFT
Random	98364.36	3517.51	88890.60	1756.48	81988.60	2191.97	17.36	8.86
Hotspot	74501.23	1124.97	67111.00	2089.43	63361.25	909.52	15.26	6.74
Transpose	74946.65	1671.31	62364.20	2694.13	50923.78	1709.00	33.82	19.45
Shuffle	68689.87	2816.28	58142.20	959.35	49478.56	1215.37	28.91	15.82
Neighbor	75090.56	412.25	66895.00	1605.48	57749.10	1001.16	27.43	14.43
Average Network Energy Consumption (uJ/Packet)								
Applications	Energy	Error ±	Energy	Error ±	Energy	Error ±	H-SMBFT % savings as compared to BFT	H-SMBFT% savings as compared to SMBFT
Random	542.32	16.14	489.73	22.26	457.56	16.20	16.65	7.46
Hotspot	428.78	12.76	389.65	17.71	367.23	17.09	15.19	6.73
Transpose	474.24	6.30	382.65	17.40	323.91	11.63	32.71	16.83
Shuffle	398.76	17.32	351.39	15.97	301.39	14.84	26.76	15.36
Neighbor	543.78	11.49	467.10	21.23	410.50	17.75	24.63	13.61
Average Network Latency (Cycles)								
Applications	Latency	Error ±	Latency	Error ±	Latency	Error ±	H-SMBFT% savings as compared to BFT	H-SMBFT% savings as compared to SMBFT
Random	1401.24	88.58	1267.35	32.92	1196.03	50.69	15.93	6.16
Hotspot	1091.62	35.08	954.96	34.87	877.37	10.91	19.75	9.34
Transpose	953.23	47.23	851.26	22.11	789.35	45.27	18.51	8.75
Shuffle	821.32	53.73	722.24	53.20	625.00	19.55	24.65	14.53
Neighbor	761.95	18.57	602.94	32.70	502.51	12.18	35.63	17.36

**Fig 3:**
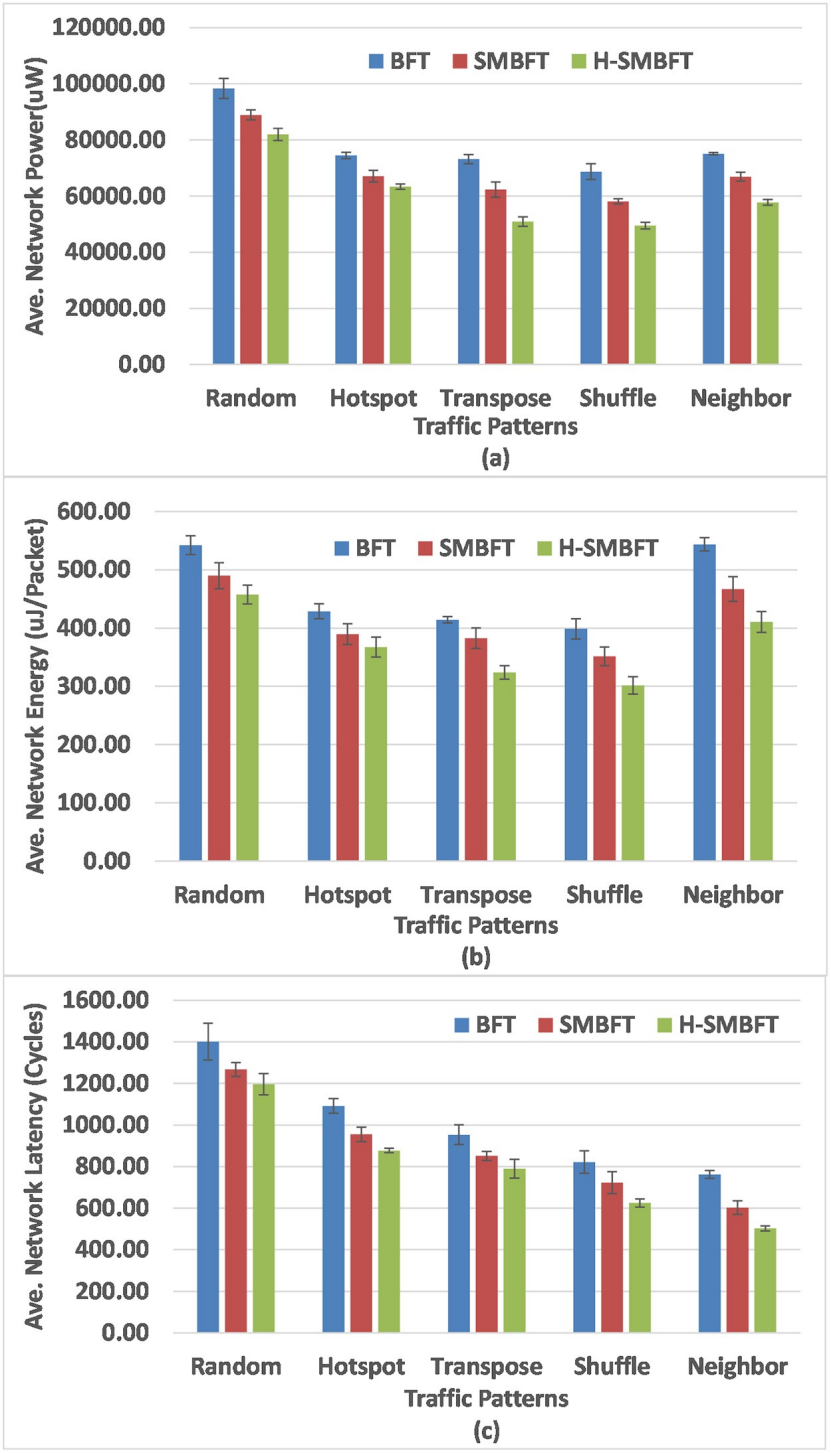
Results for synthetic traffic patterns using NoCTweak simulator. (a) Total network power, (b) Energy per data transferred packets, (c) Average network latency.

Fig 3 depicts the results of network power, energy per packet and average latency for all the topologies under consideration. In the case of Random traffic trace as shown in Table 4 and Fig 3(a)–3(c), the H-SMBFT topology gives 17.36%, and 8.86% improvement in power consumption, 16.65%, and 7.46% improvement in the energy consumption, and 15.93% and 6.16% improvement in the average network latency as compared to BFT and SMBFT respectively. Similarly, the power consumption improvement for Hotspot traffic is 15.26%, and 6.24%, the energy consumption improvement is 15.19%, and 6.73% and the average network latency improvement is 19.75%, and 9.34% compared to BFT and SMBFT respectively. For Transpose traffic, it delivers 33.82% power savings than BFT topology and 19.45% improvement over SMBFT network and the reduction in the energy consumption is 32.71% and 16.83% as compared to BFT and SMBFT networks. The H-SMBFT has 18.51% and 8.75% saving in average latency than BFT and SMBFT networks under the Transpose traffic trace. The reduction in the power consumption is 28.91% and 15.82%, the energy savings are 26.76% and 15.36% and average latency improvement is 24.65% and 14.53% as compared to BFT and SMBFT networks in the case of the shuffle traffic pattern. The H-SMBFT under the Neighbor traffic saves the power of about 27.43% and 14.6743%, lowers energy consumption of 24.63% and 13.61%, and improves average latency of 35.63% and 17.36% compared to BFT and SMBFT as detailed in Table 4 and shown in Fig 3(a)–3(c). The average network power, energy and latency error estimation is performed using the five seed t-student test. Table 4 and Fig 3(a)–3(c) (top of the bars) show the results of error estimation using five seed t-student test for H-SMBFT, SMBFT and BFT network topologies.

### 4.4 Embedded applications

The NoCTweak simulator provides a variety of real-world embedded application workloads. Table 5 lists the number of cores and the required number of streams for selected applications. We mapped all applications onto a network of 64 cores using Near-Optimal Mapping (NMAP) supported by NoCTweak. We applied for this task the very same mapping strategies as reported in [17]. Further, we used the source routing algorithm integrated into NoCTweak to compute the shortest path between all pairs of sources and destinations.

**Table 5:** Some selected real time embedded applications.

Applications	Description	Cores	Parallel Streams
DVOPD	Dual-VOPD	32	Two
Wifirx	WiFi baseband receiver	25	Two
Mpeg4	MPEG4 decoder	12	Five
Cavlc	H. 2 4 CAVLC encoder	16	Four
Telecom	E3S telecom benchmark	30	Two

**Fig 4:**
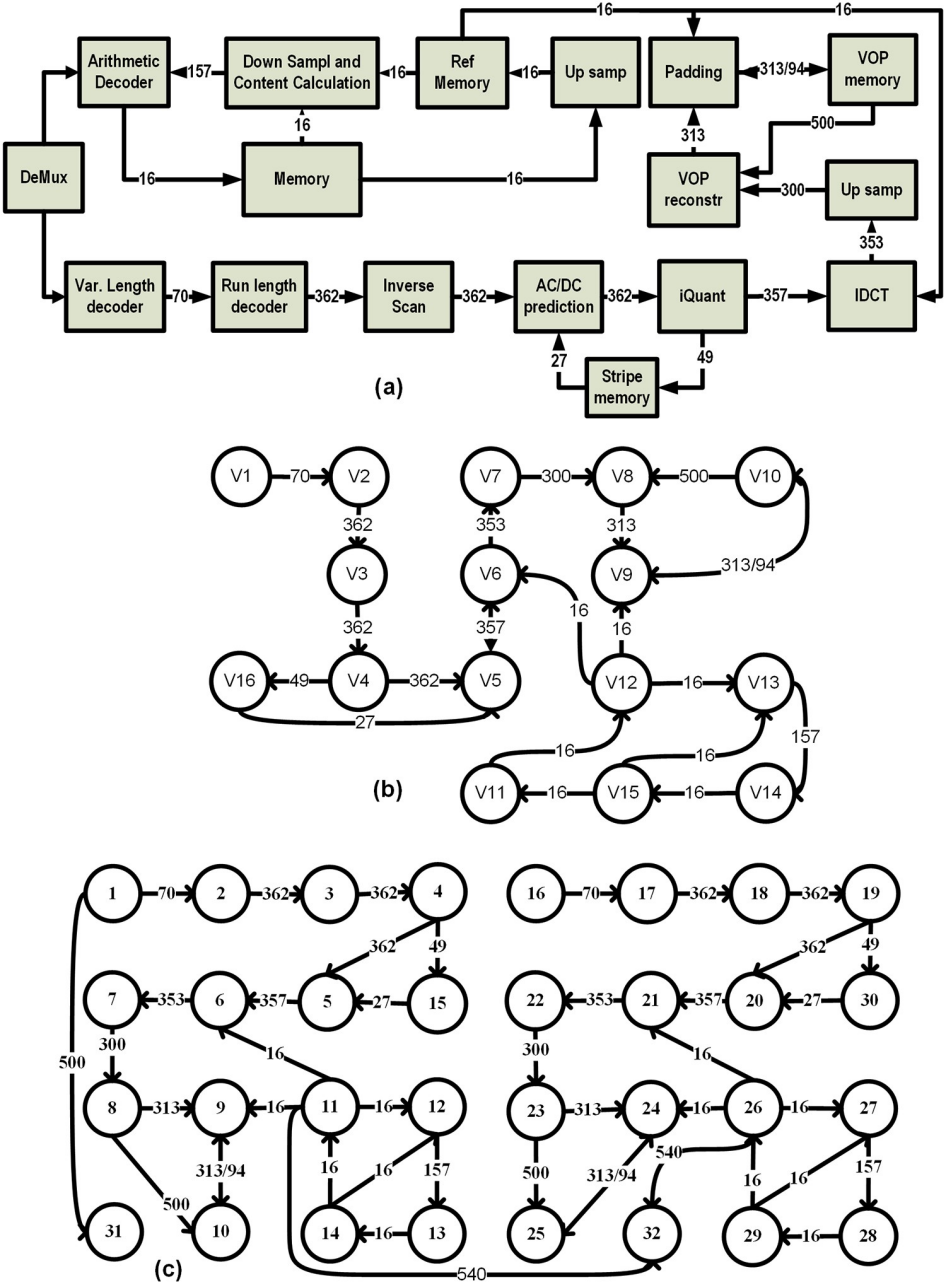
a) Block Diagram of Video Object Plane Decoder (VOPD) and, b) Core graph of VOPD, (c) Core-graph of Dual Video Object Plane Decoder. Numbers inside the circles indicate the core, while numbers on the links indicate the required bandwidth in MB/s.

The comparative analysis of BFT, SMBFT and the proposed H-SMBFT shall be illustratedwith the help of the Dual Video Object Plane Decoder (DVOPD) application workload [13]. Here, two video streams are decoded in parallel by utilizing 32 cores. This application is a scaled version of the Video Object Plane Decoder (VOPD), which consists of 16 cores (see Fig 4(a)) [13]. Each core is represented by a unique number given in parenthesis: Variable Length decoder (1), Run Length decoder (2), Inverse scan (3), AD/DC prediction (4), Iquant (5), IDCT (6), Up Sampling (7) VOP reconstructs (8), Padding (9), VOP Memory (10), Up Sampling (11), Reference memory (12) Down Sampling and Content Calculation (13), Arithmetic Decoder (14), Memory (15), Stripe memory (16).Fig 4(b) depicts the related core graph of the VOPD. The communication characteristics with uni/bidirectional links and required bandwidth in MB/s between different cores of the DVOPD benchmark are shown in Fig 4(c).

**Fig 5:**
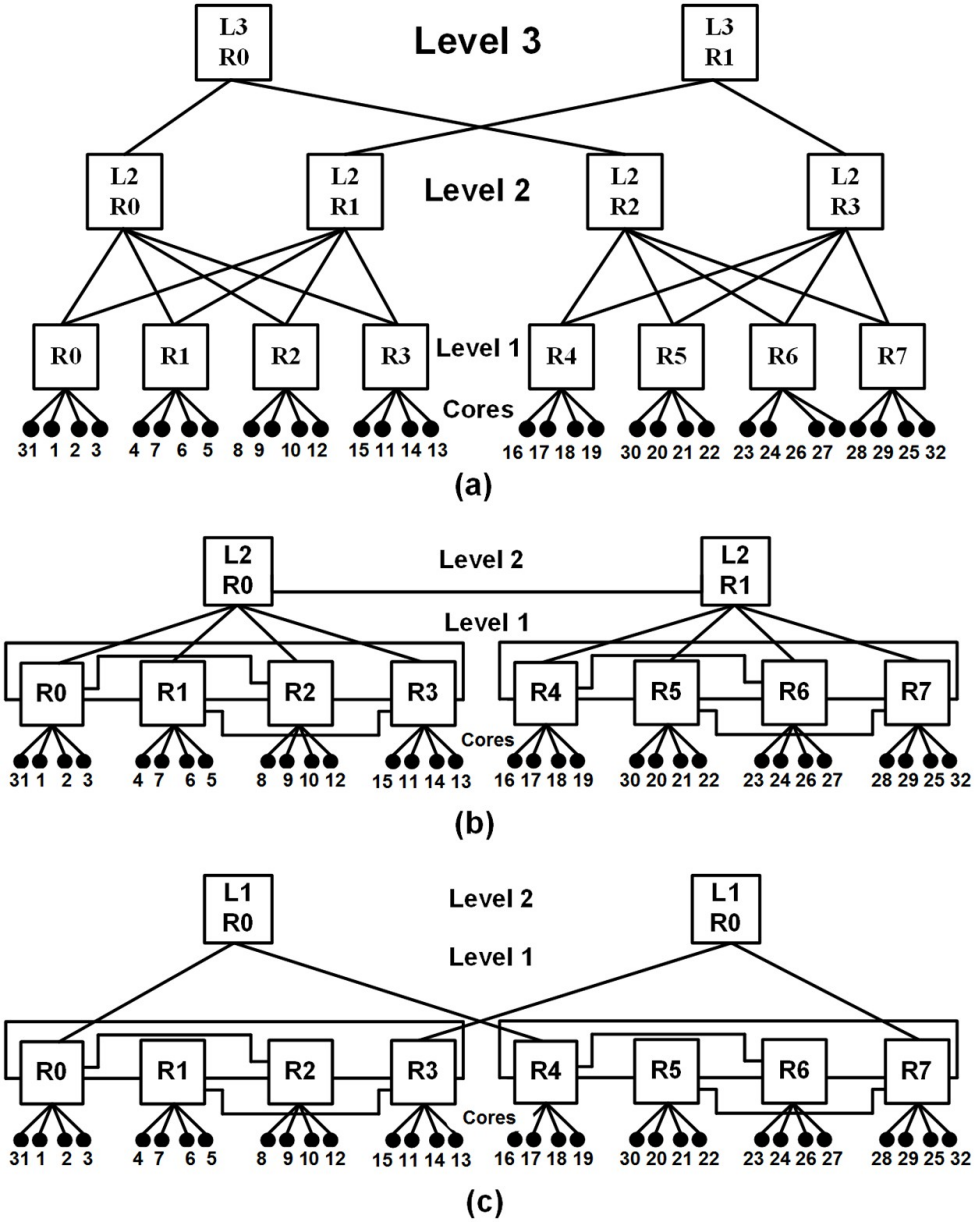
DVOPD application mapped to networks consisting of 32 cores using the topologies (a) BFT, (b) SMBFT and (c) H SMBFT.

The results of the mapping of the DVOPD using NMAP algorithm on the selected network topologies BFT, SMBFT and H SMBFT are shown in Fig 5.

All cores communicate amongst each other via routers. The communication between cores connected to the same router will take one hop. If the cores are linked to different routers, then the length of the communication path increases accordingly to the topology of the network. For example, the communication C1 → C2 between cores C1 and C2 requires all three topologies one hop via router R0 (see Fig 5). In case of the communication C3 → C4, the shortest path in the topologies SMBFT and H SMBFT involves the two level 1 router R0 and R1 (see Fig 5(b) and 5(c)). In contrast, in the topology, BFT the same communication requires the data to traverse the level 1 and level 2 routers R0, L2 R0 and R1 (see Fig 5(a)).

The longest communication path in all topologies is C11 → C32. In the case of BFT, this communication involves three levels of routers, i.e., R3 → L2R1→ L3R1→ L2R3 → R7 (see Fig 5(a)). In comparison, SMBFT requires the same communication only two levels, and the number of hops (R3 → L2R0 → L2R1 → R7) reduces from 5 to 4 (see Fig 5(b)). However, the proposed H-SMBFT requires only three hops for the same communication between C11 → C32 (R3→ L1R0→R7, see Fig 5(c)).

**Fig 6:**
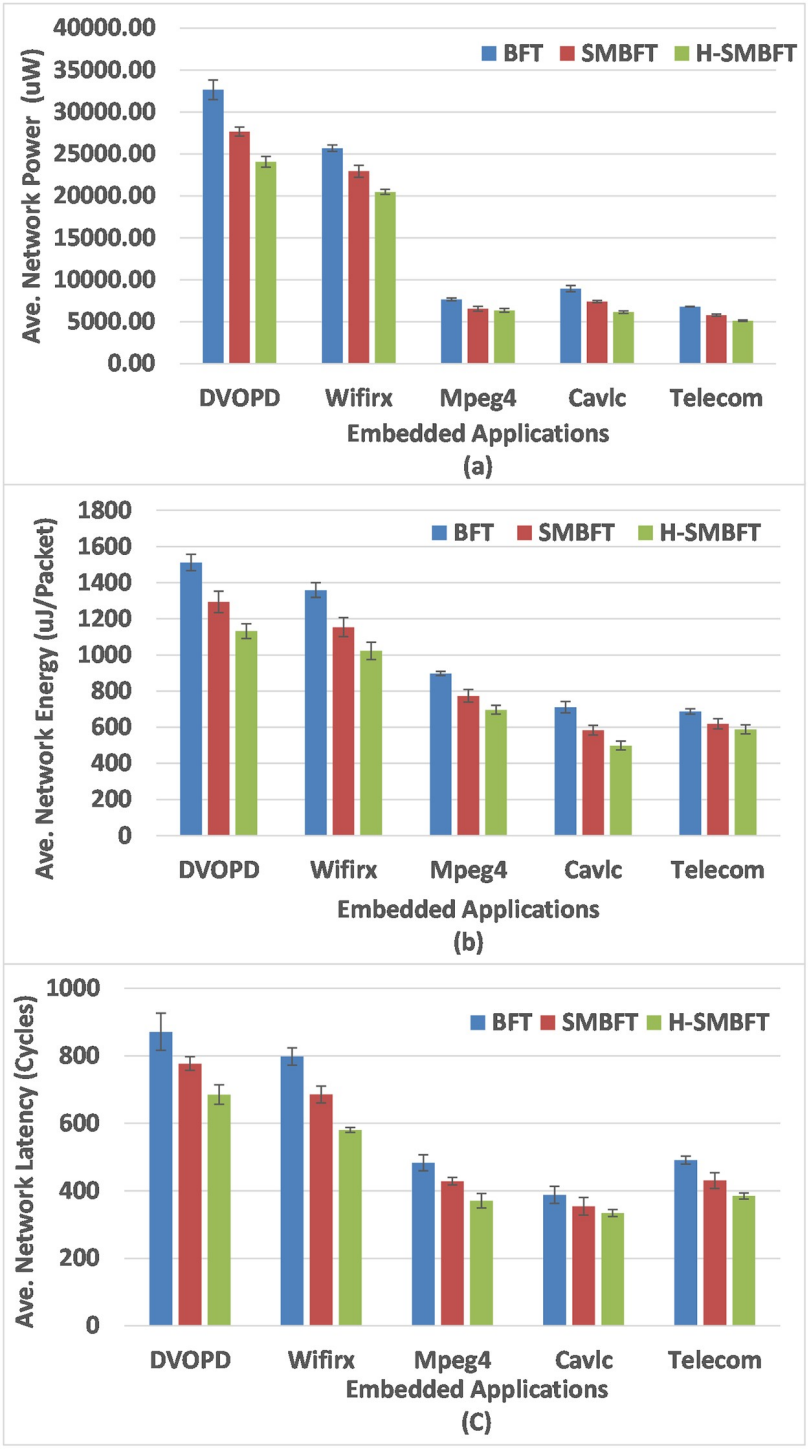
The comparison of network power, energy per packet and average network latency of competitor topologies for five different embedded application workloads using NoCTweak simulator. (a) Total network power, (b) Energy per data transferred packets and (c) Average network latency.

The results are shown in Table 6 and depicted in Fig 6(a)–6(c) in terms of Network Power, Energy and Average Latency Improvements with 64 nodes of H-SMBFT against BFT and SMBFT networks for five different real time embedded applicationworkloads using the NoCTweak simulator.

**Table 6:** Network Power, Energy and Average Latency Improvements with 64 nodes of H-SMBFT against BFT and SMBFT networks for five different real time embedded application workloads using the NoCTweak simulator.

Networks	BFT		SMBFT		H-SMBFT			
Average Network Power Consumption (uW)								
Applications	Power	Error ±	Power	Error ±	Power	Error ±	H-SMBFT% savings as compared to BFT	H-SMBFT% savings as compared to SMBFT
DVOPD	32654.10	1167.71	27656.20	546.49	24056.30	643.15	27.10	14.81
Wifirx	25689.80	387.92	22942.20	714.28	20478.20	293.96	21.23	11.71
Mpeg4	7664.63	170.92	6544.6	282.73	6353.2	213.21	18.45	8.94
Cavlc	8941.73	366.61	7412.3	122.30	6153.2	151.14	32.91	17.21
Telecom	6813.52	37.41	5789	138.94	5130.1	88.94	25.13	12.39
Average Network Energy Consumption (uJ/Packet)								
Applications	Energy	Error ±	Energy	Error ±	Energy	Error ±	H-SMBF % savings as compared to BFT	H-SMBFT % Savings as compared to SMBFT
DVOPD	1511.13	44.98	1293.8	58.82	1131.8	40.07	26.32	13.62
Wifirx	1358.92	40.45	1153.4	52.43	1022.5	47.58	25.65	12.36
Mpeg4	897.32	11.93	773.73	35.17	697	25.02	23.72	10.65
Cavlc	711.14	30.88	584.38	26.57	498.6	24.55	30.89	15.72
Telecom	687.63	14.53	619.1	28.14	588.5	25.44	24.63	11.61
Average Network Latency (Cycles)								
Applications	Latency	Error ±	Latency	Error ±	Latency	Error ±	H-SMBFT% savings as compared to BFT	H-SMBFT% savings as compared to SMBFT
DVOPD	871.32	55.08	777.15	20.18	685.26	29.04	22.34	12.61
Wifirx	797.98	25.65	685.63	25.03	580.32	7.22	28.75	16.94
Mpeg4	483.23	23.94	428.39	11.13	370.81	21.27	24.51	14.35
Cavlc	387.78	25.37	354.22	26.09	334.43	10.46	14.23	6.53
Telecom	491.21	11.97	430.65	23.36	384.65	9.32	22.73	11.32

It can be concluded from the results in Fig 6(a)–6(c) and Table 6 that in the case of two parallel streams of DVOPD application mapped on the proposed H-SMBFT and the competitors topologies BFT and SMBFT, the H-SMBFT topology gives 27.10%, and 14.81% improvement in power consumption, 26.32%, and 12.61% improvement in the energy consumption, and 22.34%, and 12.61%average network latency improvement as compared to BFT and SMBFT respectively. Power consumption improvement for two Wifirx embedded application workloads is 21.23%, and 11.71%, the energy consumption improvement is 25.65%, and 12.36% and the average network latency improvement is 28.75%, and 16.94% as compared to BFT and SMBFT respectively. The H-SMBFT, for five parallel streams of Mpeg4 workloads, saves 18.45% power than BFT topology and 8.94% power over SMBFT network and the reduction in the penalty of energy consumption is 23.72% and 10.65% as compared to BFT and SMBFT networks respectively. The H-SMBFT also improves 24.51% and 14.35% average latency than BFT and SMBFT networks, respectively. The reduction in the power consumption is 32.91% and 17.21%, the energy savings are 30.89% and 15.72% and improved performance in terms of average latency is 14.23% and 6.53% as compared to BFT and SMBFT networks under the four parallel streams of Cavlc application workloads. Similarly the H-SMBFT in the in the case of two parallel streams of Telecom application workloads, saves powerof25.13% and 12.39%, lowers energy consumption of24.63% and 11.61%, and lowers average latency of 22.73% and 11.32% compared to BFT and SMBFT as shown in Table 6 and Fig 6(a)–6(c). The average network power, energy and latency error estimation were also performed using the five seed t-student test for H-SMBFT, SMBFT and BFT networks under embedded application workloads and are shown in Table 6 and Fig 6(a)–6(c) (top of the bars).

## Results and discussion

The H-SMBFT network topology for on-chip communication is compared to its predecessors SMBFT and BFT topologies. The results show that the proposed topology can reduce the average distances as compared to SMBFT and H-SMBFT networks. The theoretical values in Table 1 show that H-SMBFT can reduce the average distance by 5.2% and 21.4% compared to SMBFT and BFT respectively. The proposed topology also has lower demands in terms of a number of links and router complexities that in turn leads to reduced costs and improved communication performance. Table 2 compares the required links and routers of BFT, SMBFT and H-SMBFT networks with 64 nodes. The results indicate that H-SMBFT manages to reduce the power consumption by 9.8% and 17.3% and improves area utilization by 7.9% and 21.1% as compared to SMBFT and BFT networks topologies respectively. Further, the proposed topology is fairly compared to its predecessor topologies by applying both the synthetic as well as real-time embedded application workloads in terms of average latency, costs, power and energy consumption of the networks.

The simulation results of Table 4 and Fig 3(a)–3(c) indicate that H-SMBFT is an efficient candidate compared to its predecessor’s topologies, with notable improvements in average latency and costs related to power and energy consumption of the network. The simulation results under five different synthetic traffic traces prove that H-SMBFT can reduce the average latency by up-to35.63% and 17.36%compared to BFT and SMBFT, respectively. The power dissipation of the network with the same number of nodes is improved by up-to 33.82% and 19.45%, and also the energy consumption of the network is improved by up-to32.71% and 16.83% compared to BFT and SMBFT, respectively. Similarly, the simulation results of Table 6 and Fig 6(a)–6(c) under five different real time embedded workloads also indicate that H-SMBFT can effectively reduce the average latency by up-to28.75% and16.94%compared to BFT and SMBFT, respectively. The power dissipation of the network is improved by up-to32.91% and 17.21%, and the energy consumption of the network is also improved by up-to30.81% and 15.72% compared to BFT and SMBFT, respectively.

## Conclusions

This work presents the indirect Hybrid Scalable-Minimized-Butterfly-Fat-Tree (H-SMBFT) network topology for on-chip communication. Compared to its predecessors, the proposed topology has lower demands regarding the number of links and router complexity that leads to reduced costs and improved communication performance. The H-SMBFT network has not only inherited the good symmetry of SMBFT and BFT networks, but it also possesses improved scalability. The results of Tables 1 and 2 show that the proposed H-SMBFT topology reduces the average distance by 5.2% and 21.4%,and lowers the demands of number of links and router complexity by 9.8% and 17.3% and also improves area utilization by 7.9% and 21.1% as compared to SMBFT and BFT networks respectively. Simulation results based on post-layout data for both the synthetic and real-world applications workloads in Tables 4 and 6, Figs 3 and 6 indicate that H-SMBFT can effectively reduce the average latency by up-to17.36% compared to SMBFT and even by up-to35.63% compared to BFT topology. Further, costs in terms of power and energy are reduced by 19.45% and 16.83% compared to SMBFT and even higher that is 33.82% and 32.91% compared to BFT topology. It is evident from the results that for all selected traffic applications the proposed H-SMBFT has the shortest average network latency and lowest costs in terms of power consumption and energy per flit compared to its competitors SMBFT and BFT. Therefore, it can be concluded that the proposed indirect H-SMBFT topology is best among its predecessors under different type of synthetic and real application traffic patterns for on-chip communication.
